# Monitoring diacylglycerols in biofluids by non-isotopically paired charge derivatization combined with LC-MS/MS

**DOI:** 10.3389/fchem.2022.1062118

**Published:** 2022-11-24

**Authors:** Yang-Dan Liu, Hua-Jun Liu, Guan-Wen Gong

**Affiliations:** ^1^ State Key Laboratory of Natural Medicines, Department of Chinese Medicines Analysis, China Pharmaceutical University, Nanjing, China; ^2^ Department of General Surgery, Affiliated Hospital of Nanjing University of Chinese Medicine/Jiangsu Province Hospital of Chinese Medicine, Nanjing, China

**Keywords:** diacylglycerols, charge derivatization, UPLC-MS/MS, quantitation analysis, acute pancreatitis

## Abstract

Diacylglycerols (DAGs) are important lipid mediators in cellular signaling transduction and metabolism. Imbalanced production or consumption of DAGs has a negative impact on the physiological functions of the body. However, comprehensive monitoring of structurally diverse DAGs remains a daunting task due to the rapid metabolism and ion suppression characteristics in biofluids. These bottlenecks call for developing a method that enables sensitive quantification of DAGs in biological sample. In this work, a straightforward charge derivatization strategy was developed to insert a series of structure analogs charge label, i.e., N, N-dimethylglycine (DMG) and N, N-dimethylalanine (DMA), on the free hydroxyl group of the DAGs. Owing to the existence of tertiary amino groups in charge label, the mass spectrometry ionization response of the derivatized DAGs was significantly increased in comparison with traditional metal ion adducts. After charge derivatization, the specific neutral loss diagnostic ions (DMG, 103 Da and DMA, 117 Da) were captured by mass spectrometry. Then, the DMG/DMA-oriented paired multiple reaction monitoring methods based on the characteristic diagnostic ions of the derivatized DAGs have been developed as sensitive methods for the detection (detection limit = 16 aM) and quantification (quantification limit = 62.5 aM) of DAGs in serum. Moreover, the tagged 1,2-DAGs and 1,3-DAGs *sn*-isomers have been well separated on the reversed-phase column in combination with ultra-performance liquid chromatography. Finally, metabolic characterizations of the tagged DAGs were further explored in L-Arginine-induced acute pancreatitis mice and resveratrol treated model mice. The results indicated that 1,2-DAGs were increased in the serum of model mice relative to normal controls and resveratrol significantly altered this metabolic abnormality. The currently established DMG/DMA-oriented paired charge derivatization strategy is promising for depicting DAGs changes more accurately in metabolic studies of lipid-related diseases and accurately evaluating drug treatment strategies.

## 1 Introduction

Neutral lipids participate in numerous metabolic pathways and play a key role in cellular energy supply, lipid synthesis and cellular signaling (Bechara et al., 2015; Shatz et al., 2016; Thiele et al., 2019). As an important subclass of neutral lipids, diacylglycerols (DAGs) are metabolites of the lipolytic process (Walther and Farese, 2012) and vital second messengers (Liu et al., 2014). In the presence of different lipases (Zechner et al., 2017; Olzmann and Carvadho, 2019), DAGs are also biosynthetic precursors of triacylglycerols (TAGs) (Olzmann and Carvadho, 2019) or glycerophospholipids (GPLs) (Wolfgang, 2021). Given its central role in multiple physiological processes, quantitative monitoring of variations in bioactive lipids is critical to reveal metabolic mechanisms.

In general, DAGs possess a characteristic constitution, where the two hydroxyl groups on the main chain of glycerol are replaced by fatty acyl groups *via* ester bond (Carrasco and Mérida, 2007). Notably, various fatty acids and different esterification positions result in versatile structures of DAGs, providing significant diversity and complexity of DAGs (Goi and Alonso, 1999; Marquez and Blumberg, 2003; André et al., 2013). Lipid metabolizing enzymes can distinguish various regional isomers of DAGs, which implies that regional isomers of DAGs have different physiological functions (Goi and Alonso, 1999; Carrasco and Mérida, 2007; Wang et al., 2016; Choi et al., 2021). In lipolysis process, adipose triglyceride lipase selectively hydrolyzes TAGs at *sn*-2 position to generate 1,3-DAGs at the lipid droplet (Carrasco and Mérida, 2007; Eichmann et al., 2012). In GPLs metabolism, phospholipase C specifically hydrolyzes GPLs at *sn-*3 position to generate 1,2-DAGs at the plasma membrane (Bunney and Katan, 2006; Li et al., 2010). The accumulation of 1,2-DAGs is relevant to activate protein kinase C (PKC) with a concentration-dependent manner, which ultimately influences intracellular Ca^2+^ releasing (Lyu et al., 2020) On the contrary, 1,3-DAGs play a much less important role in the activation of PKC compared to 1,2-DAGs. Therefore, to monitor the dynamic alterations of DGAs is essential to clarify the intersection point between lipid and signaling metabolism, which has been proved to decide different cell fates (Alwarawrah et al., 2016; Jayasinghe et al., 2019). Unfortunately, DAGs are always neglected due to the rapid metabolism in biological fluids and the ease of suppression during the measurement process.

In the case of DAGs detection, gas chromatography coupled with mass spectrometry is a conventional employed technology (Zllner, 1997). This analysis strategy firstly separates the DAGs and subsequently derived them which raises the volatility of the DAGs (Li et al., 2007). However, comparatively larger sample volumes and longer analysis times are required in the entire process. In recent years, high performance liquid chromatography (HPLC) technique has been widely utilized for the profiling of DAGs (Yu et al., 2018). DAGs, as well as other neutral lipids, can be well separated by the chromatography column, enabling quantitative determination of 1,2-DAGs and 1,3-DAGs isomers from biological samples (Deng et al., 2016; Palyzová and Ezank, 2020). However, direct detection of DAGs is usually obscured due to the low ionization efficiency. Moreover, since DAGs generated in circulation is usually hydrolyzed to MAG and fatty acid, it is probably not stable in plasma. Therefore, there is an urgent requirement for sensitive detection strategy to profile DAGs in biological samples.

Charge derivatization is a feasible and promising method to enhance lipid dissociation efficiency by ESI-MS system (Poad et al., 2019; Randolph et al., 2020). Both N, N-dimethylglycine (DMG) and DMG imidazolines have been suggested to be effective charged labels that target the free hydroxyl groups through ester bond formation (Jiang et al., 2010). The charge derivatized DAGs *via* DMG has facilitated shotgun lipid analysis by Han (Wang et al., 2014). The introduction of charge labels in DAGs increases the signal intensity of lipids more than the formation of metal addition ions (Johnson, 2001; Jiang et al., 2010; Hsu, 2016). Xia’ team introduce a charge-tag to unsaturated DAG by the Thiol-Ene Click Chemistry for lipid shotgun analysis (Adhikari et al., 2018). The collision-induced dissociation of charge-derived DAGs generally results in high-intensity specific label fragments, which facilitates the establishment of MS/MS reactive ion detection methods (Adhikari et al., 2018). In MS analysis, multiple DAGs internal standards are required for quantification due to the different lengths and unsaturation of the fatty acyl chains of DAGs. The use of stable isotope internal standard of each DAGs ensures high accuracy of quantitative measurement during MS analysis (Zhang et al., 2010). However, commercially available isotope internal standards of DAGs with different structures are extremely limited and expensive. An alternative method is to introduce structural analogs charge labels as one-to-one internal standard to correct variations caused by derivatization procedure and MS detection.

Herein, we reported a non-isotopically paired charge derivatization strategy couple with HPLC-MS/MS analysis for sensitive quantification of 1,2 DAGs and 1,3 DAGs in serum. DMG and N, N-dimethylalanine (DMA) selectively reacted with DAGs in mild condition and rapidly generated positive ion-specific tag fragment during ESI-MS electrospray ionization. DMA, as a charge-tag for DMG methylation, was used as one-to-one internal standards to correct for variations in the analysis. To our acknowledge, the DMG/DMA-oriented paired charge derivatization method was first proposed. In this study, synthetic DAGs standards with variable chain length and position location were utilized to optimize reaction condition and establish a quantitative MS/MS approach. This is an integrated approach that allows for parallel measurement of multiple chain lengths and positional heterogeneity of DAGs in a single run. The analytical capability of the proposed strategy was validated by monitoring DAGs in serum sample of acute pancreatitis and profiling variations of DAGs after resveratrol intervention. In light of the biological importance of DAGs in acute inflammation and numerous other pathological conditions, the presented DMG/DMA-oriented charge derivatization method is advisable for tracking the alterations of DAGs in pathological state and accurately evaluating drug treatment strategies.

## 2 Materials and methods

### Materials

All the chemical reagents and solvents were obtained from commercial sources and were utilized without further purification. N, N-dimethylglycine (DMG), N, N-dimethylalanine (DMA), N-(dimethylamino) pyridine (DMAP) and 1-ethyl-3-(3-dimethylaminopropyl) carbodiimide (EDC) were purchased from Sigma-Aldrich (St. Louis, MO, United States). DAG 18:0/18:0/0, DAG 16:0/18:1/0, DAGs 16:0/16:0/0, DAG 14:0/14:0/0, DAG 12:0/12:0/0, DAG 10:0/10:0/0 and DAG 8:0/8:0/0 were purchased from Avanti PolarLipids (Alabaster, AL, United States). DAG 18:0/0/18:0, DAG 16:0/0/18:1, DAG 18:1/18:1/0, DAG 18:1/0/18:1 were purchased from Nu-Check Prep, Inc. (Elysian, MN, United States). Chromatographic grade methanol and acetonitrile were purchased from Merck KGaA (Darmstadt, Germany). Mass spectrometry grade formic acid and other chemical reagents were obtained from Sigma-Aldrich Laboratories, Inc.

### Animals and treatments

Male healthy C57BL/6 mice were provided by China Pharmaceutical University (Jiangsu, China). All mice were housed in an air-conditioned rearing chamber with an ambient temperature of 25 ± 2°C, relative air humidity of 50% ± 10% and the cycle of light and darkness is 12 h. Water and food were allowed to be freely accessible during the entire study. Animal studies have been approved by the Animal Ethics Committee of China Pharmaceutical University. After 1 week of rearing acclimatization, mice (*n* = 40) were randomly divided into different research groups (*n* = 10). First group is blank control group, in which mice were administrated with the saline (1 h apart, i.p.). The second group was the acute pancreatitis model group, in which mice were treated with two intraperitoneal injections of L-arginine (3.0 g/kg, 1 h apart, i.p.). The third group was the low-dose resveratrol (30 mg/kg, i.g.) combined with L-arginine group, in which mice were given the previously described L-arginine treatment and were administered resveratrol twice daily for 3 days. The fourth group was the high-dose resveratrol (150 mg/kg, i.g.) combined with L-arginine group, in which mice were treated with both resveratrol and L-arginine as described above. After injecting L-arginine for 1 h, resveratrol was administered by gavage twice daily for three consecutive days. L-arginine was prepared by solubilizing it in 0.9% saline, the pH of this solution was adjusted to 7 with hydrochloric acid. After animal treatment, serum samples were collected from above four groups. Simultaneously, lungs were taken. All samples were stored in a −80°C refrigerator.

### Principle of derivatization

DAGs (0.2–200 nM), DMG (0.125 mM), DMAP (0.5 mM) and EDC (0.25 mM), were dissolved in acetonitrile and dichloromethane (ACN: CH_2_Cl_2_, 1:1). Subsequently, all reagents were respectively added into glass tube and then vortex for 1 min. After centrifuging the tubes at 2,500 rpm for 1 min, the sample in glass vessels reacts at 45°C for 60 min. The derivatization reaction was quenched by introducing 1.5 ml CH_2_Cl_2_/MeOH (1:1, v/v) mixture and 0.5 ml NH_4_OH (25 mM) into the glass tubes. After vortexing the glass tubes for 1 min, the sample was allowed to stand for 5 min. Next, collect the lower CH_2_Cl_2_ layer and blow dry with nitrogen. A modified Bligh-Dyer method was applied for extraction and desalination of the derived DAGs. Finally, 75 μl mobile phase (50% Isopropanol-50% Acetonitrile) was utilized to re-suspend extractive product for MS analysis. The above charge derivatization procedure was practiced to directly modify DAGs in biological sample.

### Derivatization of serum sample

For the preparation of biological samples, liquid-liquid extraction was performed to remove proteins and concentrate lipids. At first, 400 μl of ice-cold CH_2_Cl_2_: MeOH (2:1, v/v) and 10 μl of serum were introduced into a glass tube and the mixture was swirled for 2 min. Subsequently, 100 μl of deionized water was introduced and then the CH_2_Cl_2_ layer was collected. An amount of CH_2_Cl_2_ extract was shifted to a glass vial and the sample was evaporated until dry under N_2_ airflow. All dried samples were derivatized according to the derivatization principle described above.

### Qualitative and quantitative analysis of derivated diacylglycerols

An AB Sciex Triple Time-of-Flight 5,600 system in positive ion electrospray ionization was utilized for characterization of tagged DAGs. The quantification of DAGs derivatives was performed on Qsight LX50 UHPLC together with a Qsight 220 MS system (PerkinElmer, Waltham, MA, United States). The temperature of the UHPLC autosampler was maintained at 4°C and the sample injection volume was adjusted to 2 μl. Chromatography separation was performed on a Waters Acquity BEH C18 column (1.7 μm, 2.1 mm × 150 mm) at 55°C. The mobile phase consisted of solvent A (40% water-60% MeOH contains 5 mM ammonium acetate) and solvent B (90% isopropanol-10% acetonitrile with 0.1% formic acid). The mobile phase was delivered at a flow rate of 0.2 ml/min. The gradient program used was as follows: 10% B in 0–2 min, 10% B to 30% in 2–5 min, 30% B to 35% in 5–8 min, 35% B to 37% in 8–10 min, 37% B to 37% in 10–12 min, 38% B to 38% in 12–14 min, 38% B to 45% in 14–18 min, 45% B to 65% in 18–20 min, 65% B to 80% in 20–25 min, 80% B to 95% in 25–26 min, 95% B in 26–27 min, 10% B in 27–30 min. The operating parameters of the mass spectrometer were setup as below: dry gas value is 100, atomized gas value is 180, electrospray voltage is 5,500 V, and source temperature is 400°C.

## 3 Results and discussion

### Charge derivatization of diacylglycerols standards

**FIGURE 1:**
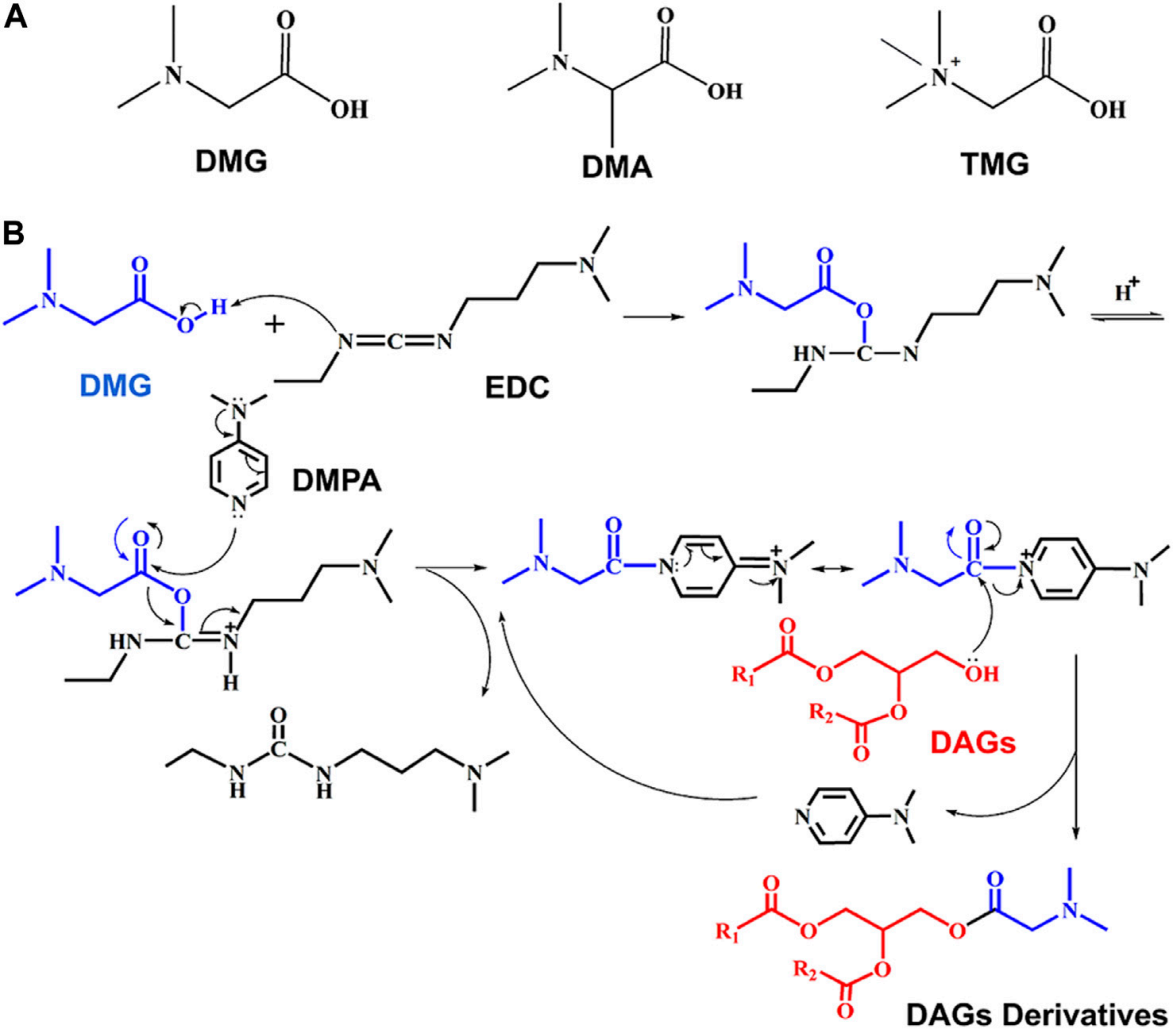
Derivatization reaction flow of DAGs. **(A)** Structure of charge derivatization reagents; **(B)** the process of charge derivatization.

Unlike polar lipid, DAGs possess relatively lower ionization efficiency under ESI-MS, because there is no fixed ionizable group in the structure. Hence, it is advisable to insert a fixed charge-tag to DAGs for enhancing ESI ionization efficiency. Three commercially available N-methylcarboxylic acid derivative reagents, comprising DMG, DMA and N, N, N-trimethylglycine (TMG), were utilized to establish the derivatization procedure (structures were shown in Figure 1A). The foundations for selecting charge derivatization reagents were functional molecules with feasible solubility and easily ionizable under ESI conditions.

Figure 1B shows a route map of derivatization (steglich esterification) reaction. In this reaction, the carboxylic acid (e.g., DMG) is firstly activated by carbodiimide. Traditionally, EDC (a carbodiimide reagent) is used to produce an O-acylisourea intermediate (Jordan et al., 2021). DMAP reacts with the alcohol on the O-acylisourea intermediate in a competitive nucleophilic manner to produce an acyl pyridinium intermediate (Xie et al., 2019). This process minimizes the generation of unproductive acyl migration side products, due to the high reaction efficiency of DMAP. The acyl pyridinium intermediate quickly reacts with hydroxyl of DAGs to reproduce DMAP and generate esterification products.

**FIGURE 2:**
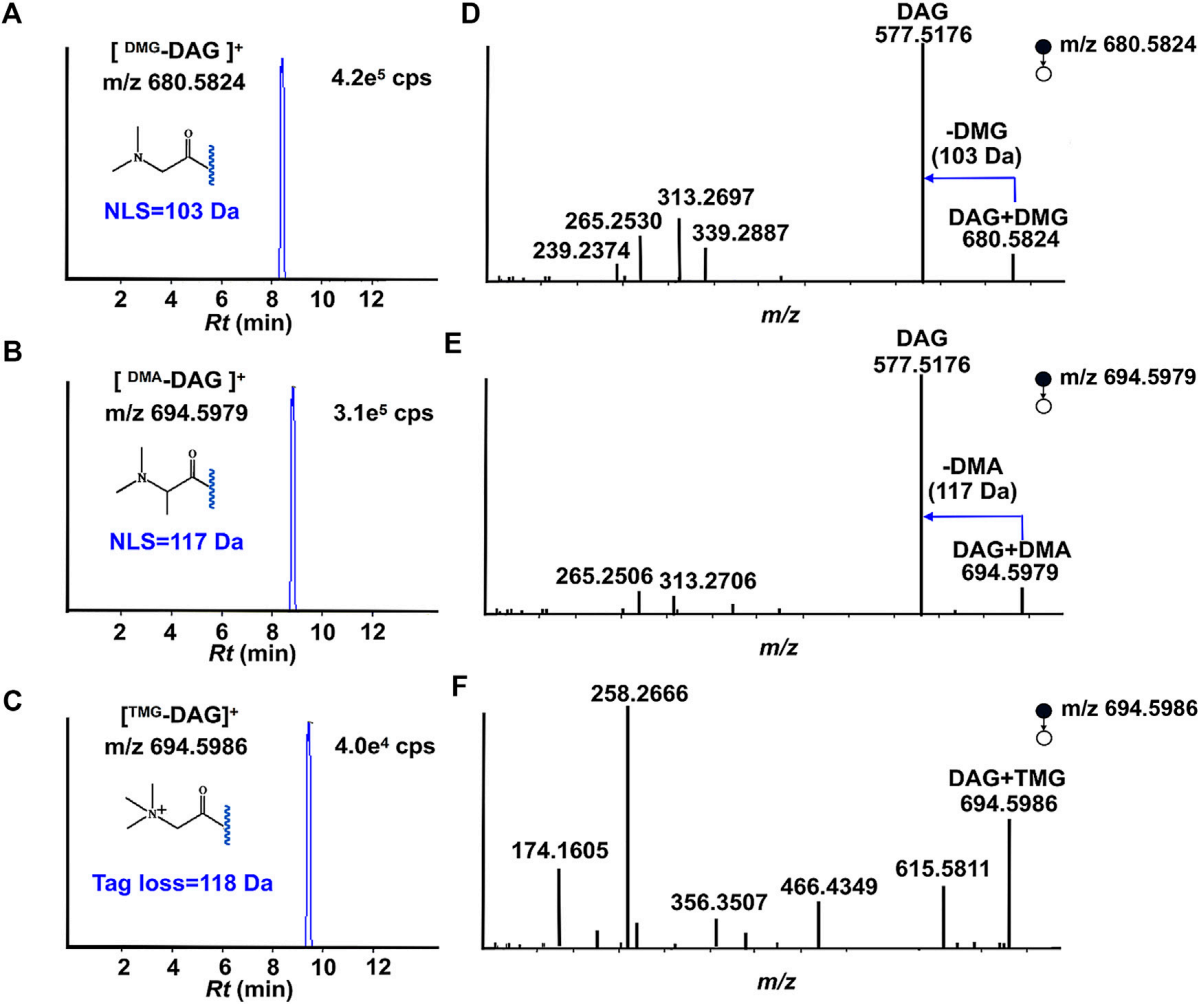
Proposed structures for fragments resulting from CID of ^Tag^-DAG. **(A,D)**: DMG; **(B,E)**: DMA; **(C,F)**: TMG.

Using DAG 16:0/18:1/0 as a model compound to verify the designed strategy. The tagged DAGs generated from three individual derivative reagents after reaching equilibrium. Figures 2A–C depict the ESI-MS spectra of DAG 16:0/18:1/0 (1 µg/ml) after the derivatization reactions. For the above derivative reagents, a single reaction product with high ionization intensity was observed, i.e., m/z 680.5824 ([DAG + DMG]^+^), m/z 694.5979 ([DAG + DMA]^+^), m/z 694.5986 ([DAG + TMG]^+^) in positive ion mode. Furthermore, all charge labeling have greatly enhanced the ionization of DAG in ESI-MS. In the case of DMG, the ionic intensity of the derived DAGs was superior to the formation of sodium adducts (Supplementary Figure S1).

The data in Figures 2D–F depict MS^2^ beam-type CID of three individual charge labeled DAGs cations in the positive ion mode. The stepwise cleavage of the DMG labeled DAGs produced a fragmentation peak at m/z 577.5176, resulting in a specific neutrality loss of 103 Da. The characteristic fragmentation signals of m/z 339.2887 and m/z 313.2697 come from the progressive loss of the fatty acyl groups of C18:1 and C16:0, respectively. Figure 2D displays the observed cleavage fragmentation information of DMG-labeled DAG 16:0/18:1/0, whereas the potential cleavage pattern is suggested in Supplementary Figure S2. In spite of the fact that all three charge labels provided better ionization at CID, DMG and DMA were selected to further develop quantitative approaches. The reason for this is that DMG- and DMA-labeled DAGs not only have characteristic fatty acyl information, the neutral loss related to the label favors the exploitation of neutral loss scan (NLS) for the determination and quantification of DAGs in biological samples. Moreover, DMA tag also introduces a stable 117 Da neutral loss. The NLS of DMA is perfectly suited for rectifying varies during detection. Instead, different from beam-type CID of TMG derivatized cation, 118 Da loss (-TMG) from derivatized cation was not observed.

**FIGURE 3:**
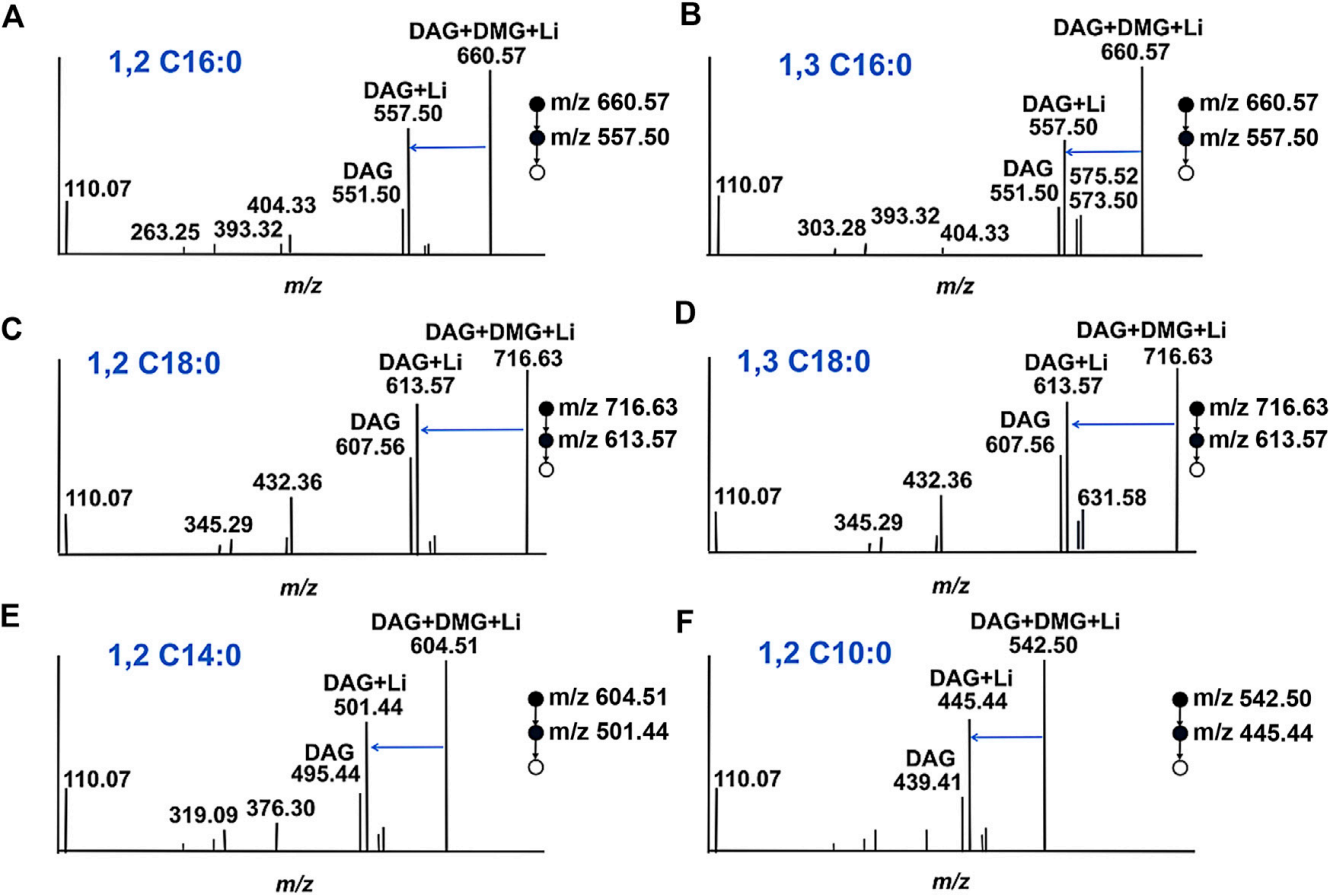
Proposed structures for fragments resulting from CID of ^DMG^-DAGs. **(A)** DAG 16:0/16:0/0; **(B)** DAG 16:0/0/16:0; **(C)** DAG 18:0/18:0/0; **(D)** DAG 18:0/0/18:0; **(E)** DAG 14:0/14:0/0; **(F)** DAG 12:0/12:0/0.

For DAGs with various chain length fatty acyl groups and different esterification positions, we wondered whether charge labeling would occur. The data in Figures 3A–F depict the derivatization spectra of DAG 16:0/16:0/0, DAG 16:0/0/16:0, DAG 18:0/18:0/0, DAG 18:0/0/18:0, DAG 14:0/14:0/0 and DAG 10:0/10:0/0, which were individually derived and performed at the same reaction conditions. The production of DMG tagging products reached a steady state during MS analysis. The fragmentation signal at m/z 557.50 is derived from DAG 16:0/16:0/0 and DAG 16:0/0/16:0, resulting from the sequential loss of the DMG tag (Figures 3A,B). Similarly, the peak at m/z 613.57 is derived from DAG 18:0/18:0/0 and DAG 18:0/0/18:0 (Figures 3C,D). Although the existence of different positional isomers in DAGs, only a single DMG-labeled product was detected. For different fatty acyl chain lengths, both DAG 14:0/14:0/0 (Figure 3E) and DAG 10:0/10:0 (Figure 3F) had DMG-labeled fragments at m/z 501.44 and m/z 445.44, respectively. The above results demonstrate that the presentation of a charge label to DAG species produces a similar ionic response in ESI-MS, no matter the length or positional heterogeneity of the fatty acyl chain. However, DMG may be unable to distinguish positional isomers in the DAG. This is because none any significant and stable diagnostic fragments were observed during ESI-MS analysis.

### Optimization of charge derivatization

On the basis of derivatization of DAGs reference standard, we proceeded to explore the applicability of charge derivatization in biological samples. Supplementary Figure S3 summarizes that fragments resulting from CID of DAG 16:0/16:0/0, DAG 18:0/18:0/0 and DAG 18:1/18:1/0 in serum samples. The DAG standards were simultaneously charge labeled by derivatization reaction which resulted in an enhancement of the MS signal. It is agreed with the above findings that polar head group of lipid has a primary effect on the dissociation of DAGs *via* ESI-MS. Different DGAs species modified with polar charge labels share basically the similar ionization efficiency at low concentrations. The Steglich esterification reaction parameters have been optimized in order to develop a feasible derivatization method that satisfies the demands of less hazardous solvent systems, shorter reaction time and mild reaction temperatures for biological samples. Previous efforts to use common green solvents (e.g., ethyl acetate and acetone) failed to produce a product (Churruca et al., 2012). The reaction is usually carried out using anhydrous chloride solvent in the presence of nitrogen. However, these solvents are undesirable because they can be hazardous to environment and human health. Hence, a combined solvent system, included acetonitrile and dichloromethane (v/v, 1:1), has been tried for derivatization.

**FIGURE 4:**
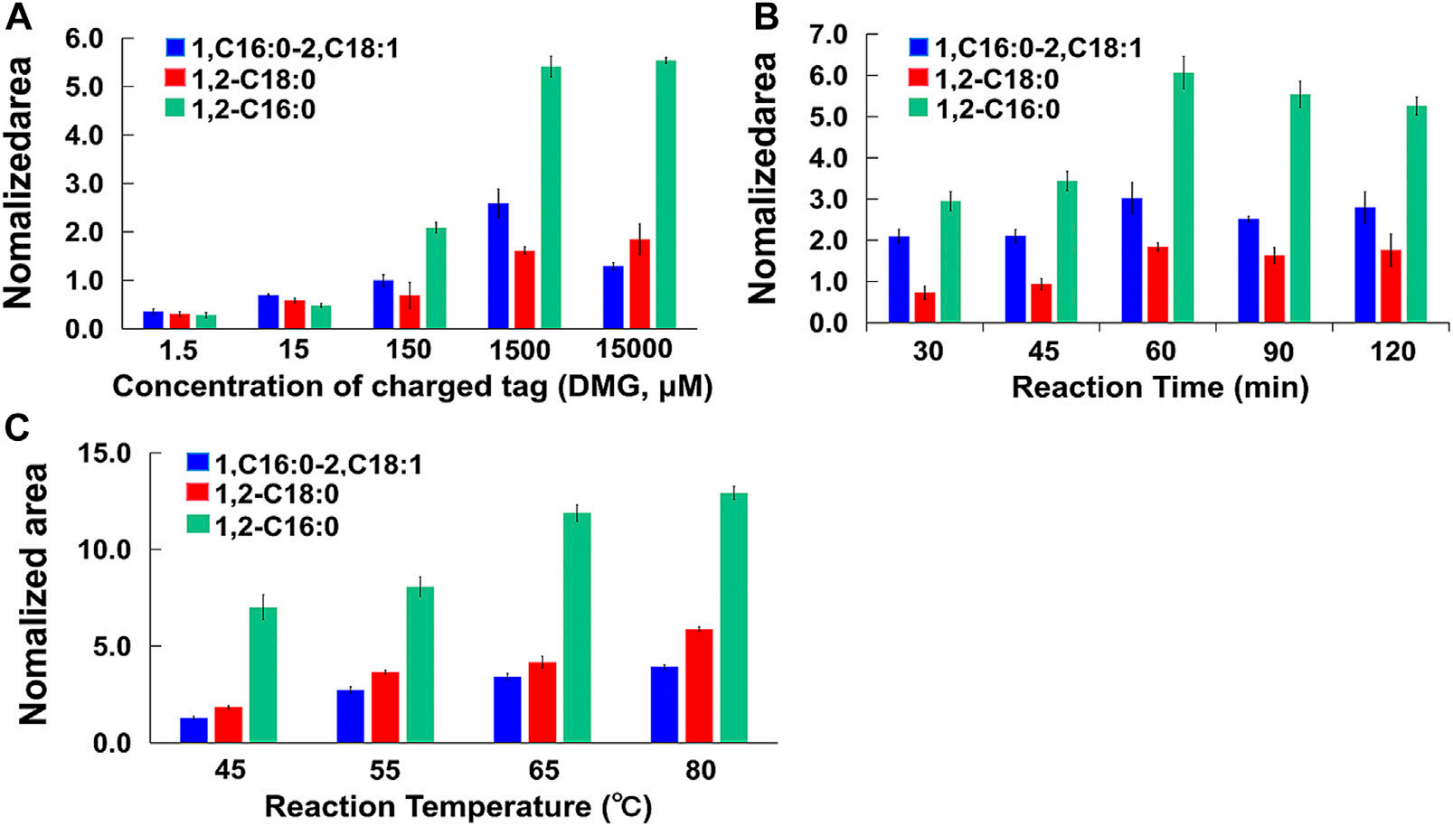
**(A)** Nomalized area of labeled DAGs standers under different concentration of charged tag; **(B)** Nomalized area of labeled DAGs standers within different reaction time; **(C)** Nomalized area of labeled DAGs standers under different temperatures.

Prior researches have pointed out that the initial nucleophilic reaction participated by DMAP is a speed-limiting step for this reaction (Figure 1). To facilitate the reaction as much as possible, the derivatization reaction was carried out with excessive amounts of DMAP (0.5 mM). Because hydroxyl groups are widely present in biological samples, a large amount of charge tags are in favor of complete derivatization. Different amount of charge tags (DMG, range from 1.5 μM to 15,000 μM) were tested to obtain optimal derivatization using DAG 16:0/16:0/0, DAG 18:0/18:0/0, DAG 16:0/18:1/0 (1.5 μM) as mixed standard. Excess DMG promoted the formation of charged derivatives. It was found that when DMG was 1,500 μM, the most DAG derivative products were generated. Interestingly, more DMG (15,000 μM) did not induce higher labeled product, suggesting DAGs have been completely derivatized (shown in Figure 4A).

Different reaction time periods (30 min, 45 min, 60 min, 90 min, and 120 min) and temperatures (45°C, 55°C, 65°C and 80°C) were tested to obtain optimal derivatization. The conditions were selected to be examined in order to keep the solvent system under boiling point and avoid using a reflux condenser, thereby ultimately obtaining a better reaction rate. Based on the MS intensities achieved from the labeled DAGs standards, the intensities of the DAGs at 60 min were higher than those at 90 min and 120 min (Figure 4B). On this basis, the intensity was higher at 80°C than at 65°C. However, we finally opted for 65°C and 60 min based on principles of avoiding unanticipated side reactions in biological system under gentle reaction condition (Figure 4C).

### Quantitative analysis and method validation

**FIGURE 5:**
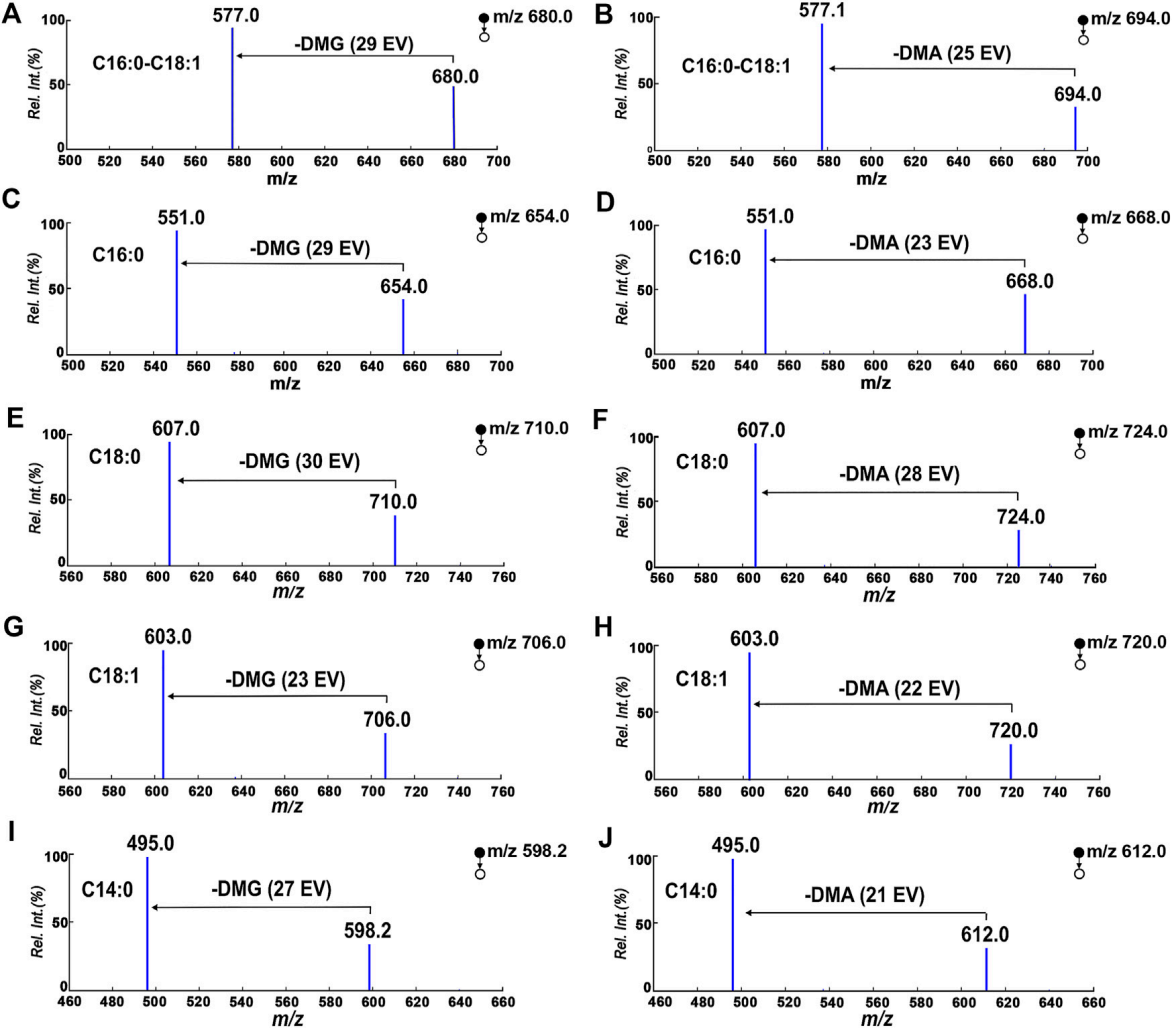
Multiple reaction monitoring beam-type CID of ^DMG^-DAGs and ^DMA^-DAGs in positive mode. **(A,B)** DAG16:0/18:1/0, **(C,D)** DAG16:0/16:0/0; **(E,F)** DAG18:0/18:0/0, **(G,H)** DAG18:1/18:1/0; **(I,J)** DAG14:0/14:0/0.

**FIGURE 6:**
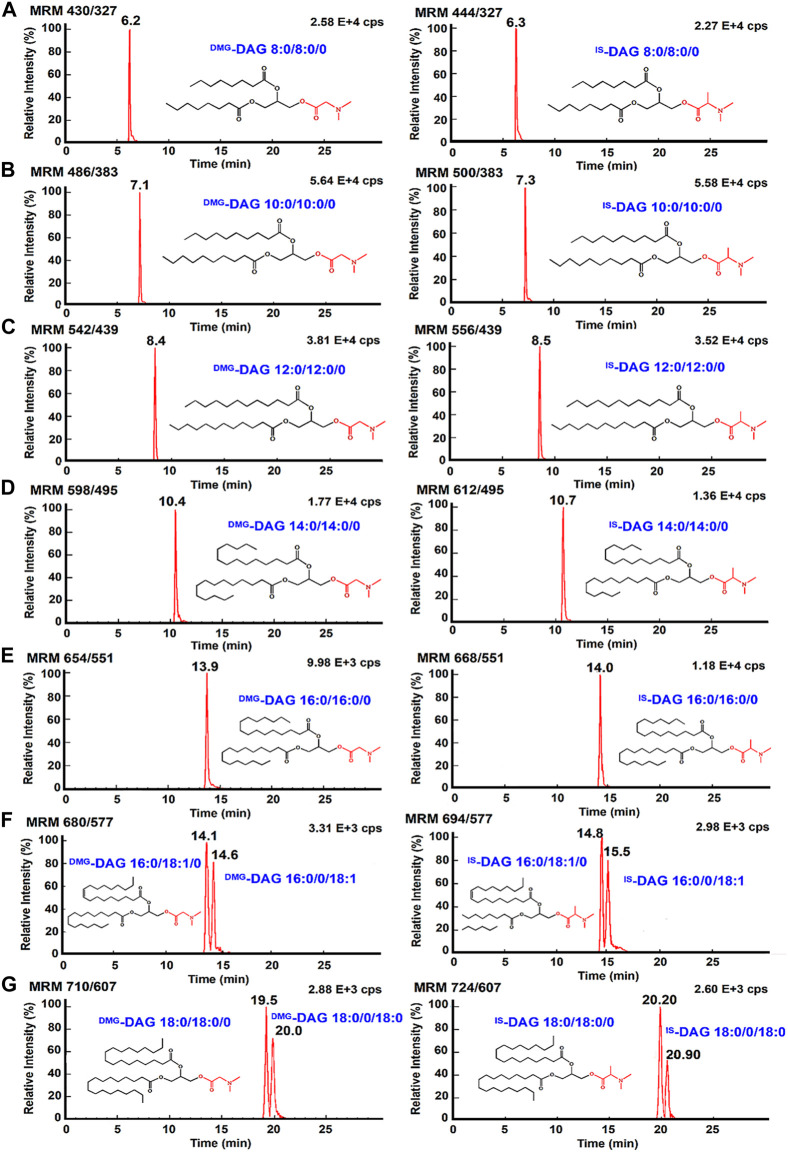
The chromatogram of DAGs, showing separation of *sn*-1,2 DAGs and *sn*-1,3 DAGs. UPLC-MS/MS chromatogram of **(A)** ^DMG^-DAG 8:0/8:0/0 and ^IS^-DAG 8:0/0/8:0; **(B)** ^DMG^-DAG 10:0/10:0/0 and ^DMA^-DAG 10:0/0/10:0; **(C)** ^DMG^-DAG 12:0/12:0/0 and ^DMA^-DAG 12:0/12:0/0; **(D)** ^DMG^-DAG 14:0/14:0/0 and ^DMA^-DAG 14:0/14:0/0; **(E)** ^DMG^-DAG 16:0/16:0/0 and ^DMA^-DAG 16:0/16:0/0; **(F)** ^DMG^-DAG 16:0/18:1/0, ^DMG^-DAG 16:0/0/18:1, ^DMA^-DAG 16:0/18:1/0 and ^DMA^-DAG 16:0/0/18:1; **(G)** ^DMG^-DAG 18:0/18:0/0, ^DMG^-DAG 18:0/0/18:0, ^DMA^-DAG 18:0/18:0/0 and ^DMA^-DAG 18:0/0/18:0.

The properties of the 103 Da tag NLS were evaluated in terms of quantification of DAGs under optimal reaction conditions. The mass spectrometry quantitative parameters were optimized in Supplementary Table S1. Multiple reaction monitoring beam-type CID of ^DMG^-DAGs and ^DMA^-DAGs were shown in Figure 5. These ion pair are m/z 710→m/z 607 for ^DMG^-DAG 18:0/18:0/0, m/z 724→m/z 607 for ^DMA^-DAG 18:0/18:0/0, m/z 680→m/z 577 for ^DMG^-DAG 16:0/18:1/0, m/z 694→m/z 577.1 for ^DMA^-DAG 16:0/18:1/0, m/z 654→m/z 551 for ^DMG^-DAG 16:0/16:0/0, m/z 668→m/z 551 for ^DMA^-DAG 16:0/16:0/0, m/z706→m/z 603 for ^DMG^-DAG18:0/18:0/0, m/z720→m/z 603 for ^DMA^-DAG 18:0/18:0/0. The chromatograms of DAGs in UPLC-MS analysis were showed in Figure 6. Both ^DMG^-DAGs and ^DMA^-DAGs possess stable and reproducible characteristic neutral loss fragment.

The charge derivatization introduced a sensitive labeling for quantitative analysis, however, the charge tag may not be able to discriminate the *sn*-1,2 DAGs from the *sn*-1,3 DAGs. A gradient elution procedure of mobile phase was performed to separate different isomers of DAGs (in Supplementary Figure S4). As shown in Supplementary Figure S5, the isomers of DAGs were well-separated at retention times of 20.9 min and 21.6 min for ^DMA^-DAG 18:0/18:0/0 and ^DMA^-DAG 18:0/0/18:0, 20.3 min and 22.9 min for ^DMG^-DAG 18:0/18:0/0 and ^DMG^-DAG 18:0/0/18:0, 14.9 min and 15.4 min for ^DMG^-DAG 16:0/18:1/0 and ^DMG^-DAG 16:0/0/18:1, respectively.

Calibration standards, quality control (QC) samples and surrogate matrices excluding DAGs were prepared for the validation of MS quantification method. A series of standard solutions at ten levels of concentration (0.1, 1.0, 5.0, 10, 25, 50, 100, 250, 500 ng/ml) were prepared by dilution of DAG stock solutions with dichloromethane. QC samples were obtained by mixing each biological extract in equal volumes. A modified Bligh and Dyer procedure was applied for preparing surrogate matrices without DAGs. At first, 10 μl of serum was shifted into a 10 ml glass pipe, followed by the addition of 400 μl of cold CH_2_Cl_2_/MeOH mixed solvent (2:1, v/v). The mixture was swirled for 2 min at 4°C and H_2_O (100 μl) was added. The obtained solvent was homogenized and then allowed to stand for 5 min. Finally, the upper layer of organic phase (250 μl) was collected and dried by evaporation to perform derivatization.

Linearity and sensitivity: Solvent calibration curve and surrogate matrix calibration curve were established by adding calibration standards in pure solvent and surrogate matrices without DAGs (in Table 1). Linearity was evaluated by correlation coefficient (*r* ^2^). The lower limit of detection (LLOD) and lower limit of quantification (LLOQ) in MS detection were used to assess the sensitivity of this method. The LLOQ should satisfy a signal-to-noise ratio (S/N) greater than 10 and a relative standard deviation (RSD) of the assay results less than or equal to 20% (*n* = 3). The lower limit of detection (LLOD) requires a signal-to-noise ratio greater than three and an RSD less than or equal to 20% (*n* = 3).

**TABLE 1:** Calibration curves and LODs for the analysis of ^DMG^-DAGs.

Analytes	Solvent-only calibration curves		DAGs-free surrogate calibration curves		LLOQ (amol)	LLOD (amol)	Linear range (amol)
	Regression line	*R* ^2^	Regression line	*R* ^2^			
1,2-C8:0	Y = 0.1313x-0.3503	0.9980	Y = 0.1372x+0.1788	0.9963	72.5	29.0	145–725000
1,2-C10:0	Y = 0.0050x+0.0029	0.9964	Y = 0.0046x+0.0086	0.9982	62.5	25.0	125–625000
1,2-C12:0	Y = 0.0043x-0.0053	0.9963	Y = 0.0040x+0.0042	0.9933	109.5	21.9	109.5–547500
1,2-C14:0	Y = 0.0057x+0.0249	0.9982	Y = 0.0055x+0.0602	0.9981	97.5	19.5	195–487500
1,2-C16:0	Y = 0.0270x+0.0074	0.9946	Y = 0.0290x+0.1953	0.9931	88.0	17.6	176–704000
1,2-C18:0	Y = 0.0051x+0.3045	0.9965	Y = 0.0051x+0.4462	0.9973	80	16.0	160–640000
1,3-C18:0	Y = 0.0022x-0.0235	0.9979	Y = 0.0025x+0.2336	0.9960	80	16.0	160–640000
1,2-C18:1	Y = 0.0064x+0.0529	0.9979	Y = 0.0053x+0.0665	0.9959	80.5	16.1	161–644000
1,3-C18:1	Y = 0.0193x-0.4013	0.9987	Y = 0.0157x-0.0327	0.9987	80.5	16.1	161–644000
1, C16:0-2, C18:1	Y = 0.0225x+0.0176	0.9943	Y = 0.0221x+0.0267	0.9967	84	16.8	168–672000
1, C16:0-3, C18:1	Y = 0.0047x+0.0522	0.9964	Y = 0.0035x+0.0318	0.9988	84	16.8	168–420000

The availability of alternative matrix curves was evaluated by comparing the slope of the alternative matrix curve and the solvent curve. The concentrations of QCs samples were calculated from both types of curves and the deviations between the profiles were evaluated. After parallelism was confirmed, three standard solutions at low, medium and high concentrations were selected for subsequent validation analysis based on the linear range of the alternative matrix curves.

The matrix effect (ME) is measured as the ratio of three concentrations of standards (neat solution samples) to standards added to the alternative matrix (matrix samples). The ME was calculated by the following equation, where R represents the detection response of the DAGs.

$$ME\;\left(\%\right)=\frac{R_{matrix}\times {IS}_{neat\;solution}}{R_{neat\;solution}\times {IS}_{matrix}}$$

Extraction recovery efficiency was measured from the concentration of standards before (A) and after extraction (B), where the concentration was calculated from the alternative matrix curve (*n* = 3, three copies of each concentration were prepared). Recovery (%) = (A/B) × 100%. Matrix effect and recovery were listed in Supplementary Table S2.

Repeatability and stability were examined by analyzing six replicates of the QC samples in parallel. Precision was investigated by intra-day and inter-day RSD, which were measured in 1 day and on three consecutive days (*n* = 6). The storage stability of the autosampler (4°C) for the derivatized DAG was inspected by testing QC samples over a 48-h period. In addition, the stability of IS at 4°C for 48 h was also assessed. As shown in Supplementary Table S3, the RSD values for all groups were less than 20%.

### Application to analysis of diacylglycerols in pathological serums

Several researches have indicated an association with enhanced levels of DAGs in acute pancreatitis. DAGs activate PKC system, which lead to the phosphorylation of cellular calcium channels. Increased intracellular calcium promotes pancreatic cell apoptosis. Recently, Shahram et al. have shed light on potential impact of resveratrol on acute pancreatitis (Agah et al., 2021). Different metabolic pathways involved in triacylglycerol metabolism have been shown to be targets for resveratrol (Churruca et al., 2012). Hence, whether resveratrol can regulate the metabolism of DAGs was explored.

Acute pancreatitis models were invited by two intraperitoneal injections of L-arginine (i.p.). The changes in the histological structure of the pancreas and lungs from the acute pancreatitis model animals were depicted by H&E staining (Supplementary Figure S6). No alterations in the histological structure of the pancreas were observed in normal control mice. Mice with L-arginine-induced acute pancreatitis exhibited obvious alterations in pancreatic histological structures, such as acinar cell atrophy, necrosis and inflammatory cell infiltration, whereas resveratrol treatment significantly diminished pancreatic atrophy and inflammation, especially in the resveratrol high-dose group. In addition, L-arginine exposure has enhanced alveolar thickening and the amount of inflammatory cell aggregation. Whereas, treatment with resveratrol significantly restored the symptoms of alveolar abnormalities. Therefore, the results suggest a potential protective effect of resveratrol on L-arginine-induced acute pancreatitis and related lung injury.

**FIGURE 7:**
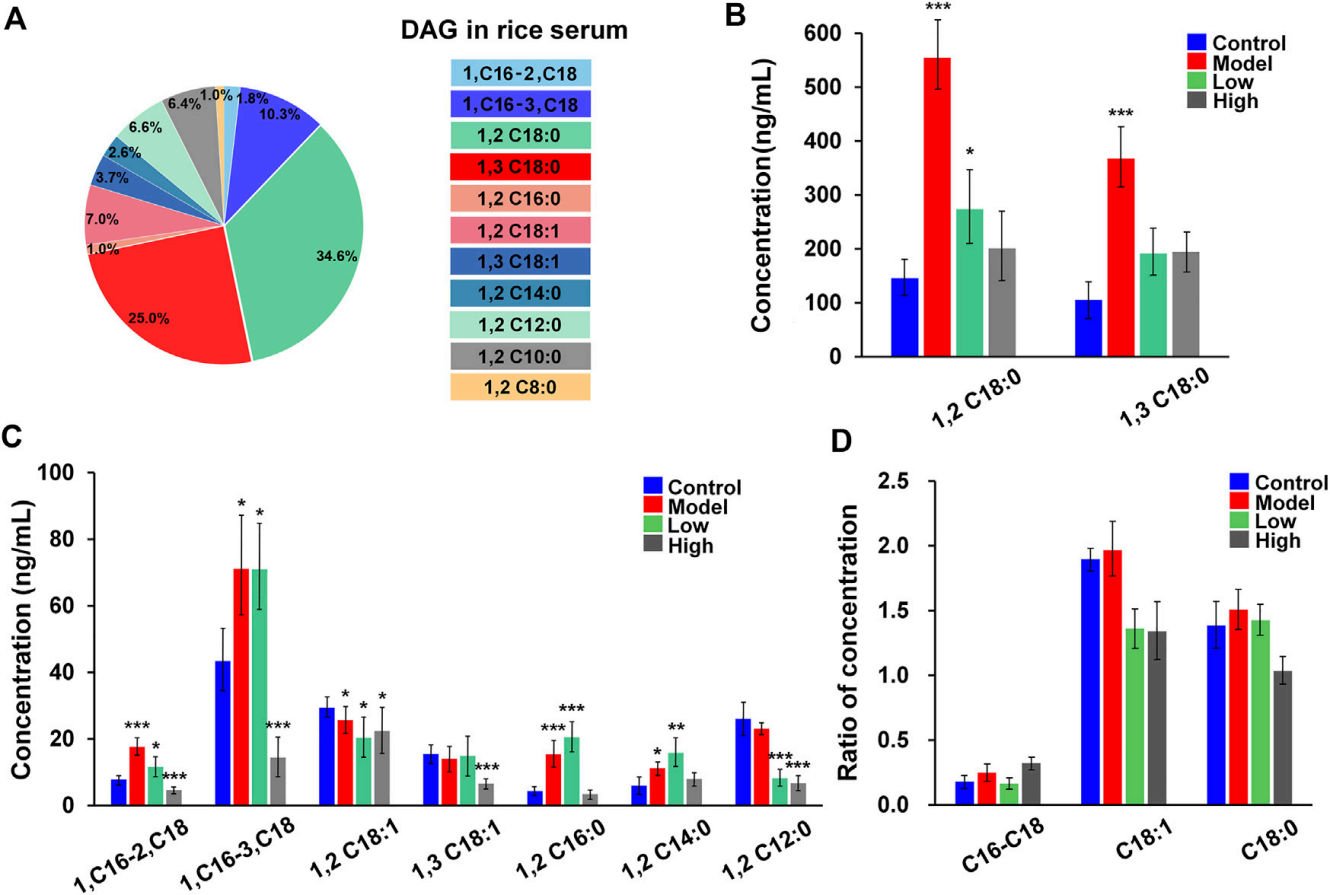
The amount of major DAGs in serum samples. **(A)** The 11 kinds of DAGs were quantified in serum samples from normal control mice; **(B,C)** The amount of DAG 18:0/18:0/0, DAG 18:0/0/18:0, DAG 16:0/18:1/0, DAG 16:0/0/18:1, DAG 18:1/18:1/0, DAG 18:1/0/18:1, DAG 16:0/16:0/0, DAG 14:0/14:0/0, DAG 12:0/12:0/0 in serum samples from normal control, acute pancreatitis mice, and resveratrol (low-dose and high-dose);. **(D)** Ratio of DAGs concentration, C16-C18 (DAG 16:0/18:1/0 vs. DAG 16:0/0/18:1), C18:1 (DAG 18:1/18:1/0 vs. DAG 18:1/0/18:1), C18:0 (DAG 18:0/18:0/0 vs. DAG 18:0/0/18:0). The *t* test has performed and *p* values were also added in **(B,C)** * indicates *p* < 0.05, ** indicates *p* < 0.01 and *** indicates *p* < 0.001.

Additionally, we were interested in checking whether the DMG label and 103 Da NLS were available for the analysis of DAGs in the serum of mice with acute pancreatitis. Figure 7A summarizes that the 11 DAG species were quantified in serums from blank control mice, they were DAG 18:0/18:0/0, DAG 18:0/0/18:0, DAG 16:0/18:1/0, DAG 16:0/0/18:1, DAG 18:1/18:1/0, DAG 18:1/0/18:1, DAG 16:0/16:0/0, DAG 14:0/14:0/0, DAG 12:0/12:0/0, DAG 10:0/10:0/0, and DAG 8:0/8:0/0. The chromatograms of DAGs were showed in Figure 6 and the concentrations of DAGs range from 4.14 ng/ml to 145 ng/ml. The DAGs *sn-*isomers, including DAG 18:0/18:0/0, DAG 18:0/0/18:0, DAG 16:0/18:1/0, DAG 16:0/0/18:1, DAG 18:1/18:1/0, and DAG 18:1/0/18:1 were separately detected. The intensity changes of DAGs from four sets of samples were summarized in Figures 7B,C (normal controls, models, low dose, high dose, *N* = 6). The content of DAG 18:0/18:0/0, DAG 18:0/0/18:0, DAG 16:0/16:0/0, and DAG 16:0/18:1/0 increased by 3.4 ± 2.1, 3.5 ± 2.0, 3.6 ± 1.5, and 2.3 ± 1.8 times in models as compared to the control, respectively. After resveratrol intervention, the levels of DAGs, including *sn*-1,2 and *sn*-1,3-DAGs, both decreased. To investigate the effect of resveratrol on positional isomers of DAGs, the ratio of *sn*-1,2-DAGs to *sn*-1,3-DAGs in different groups was analyzed. Figure 7D depict the ratios of *sn*-1,2-DAGs to *sn*-1,3-DAGs in four groups. *Sn*-1,2-DAGs, including DAG 16:0/18:1/0, DAG 18:0/18:0/0, and DAG 18:1/18:1/0, showed an elevated trend in the pancreatitis model. In the resveratrol treatment group, the ratios of *sn*-1,2-DAGs to *sn*-1,3-DAGs was decreased. This data suggests that resveratrol may have more influence on *sn*-1,2-DAGs production when regulating lipolysis. Previous studies have also revealed that resveratrol may regulate certain enzymes involved in the production of DAGs, such as phospholipase C or adipose triglyceride lipase (Yang et al., 2008; Churruca et al., 2012). In future, further studies require more sample numbers of acute pancreatitis. Based on the characteristics of the DMG charge derivatization method for amplifying MS signals, we will proceed to deeply explore the implications of structural alterations of DAGs on diseases, thereby achieving a detailed molecular-level characterization of DAGs and providing precise lipid structure information for disease research.

## 4 Conclusion

In this study, we have successfully established a strategy by pairing charge derivatization with UHPLC-MS/MS for the sensitive quantification of DAGs in biological samples. The free hydroxyl groups of DAGs were derivatized by commercially available DMG and DMA charge labels in gentle condition. The specific neutral loss diagnostic ions were captured by mass spectrometry, which allowed enhanced ionization of various DAGs in comparison with conventional metal adducts. Then, the DMG/DMA-oriented paired multiple reaction monitoring methods based on the diagnostic ions of the derivatized DAGs have been developed for the detection and quantification of DAGs in serum. The derivative 1,2-DAGs and 1,3-DAGs *sn*-isomers have been well-separated on the reversed-phase column. The proposed method was further adapted to the DAGs profiling of mice serum. Analysis of serum samples from mice with acute pancreatitis demonstrates that DAGs are found with significant changes of *sn*-1,2-DAGs in model plasma relative to normal control. Thus, the potential of the proposed the DMG/DMA-oriented paired charge derivatization method for detecting and quantifying DAGs selectively is expected to play an important role in metabolic studies of diseases associated with DAGs.

## Data Availability

The original contributions presented in the study are included in the article/Supplementary Material, further inquiries can be directed to the corresponding authors.
