# Supplementary material for: Monitoring diacylglycerols in biofluids by non-isotopically paired charge derivatization combined with LC-MS/MS
Source: Front Chem. 2022 Nov 24;10:1062118. doi: 10.3389/fchem.2022.1062118 (PMC9745812; doi:10.3389/fchem.2022.1062118)
Supplement: Supplementary file 1 [file DataSheet1.docx]

Supplementary Material

# Supplementary Figures and Tables

## Supplementary Figures


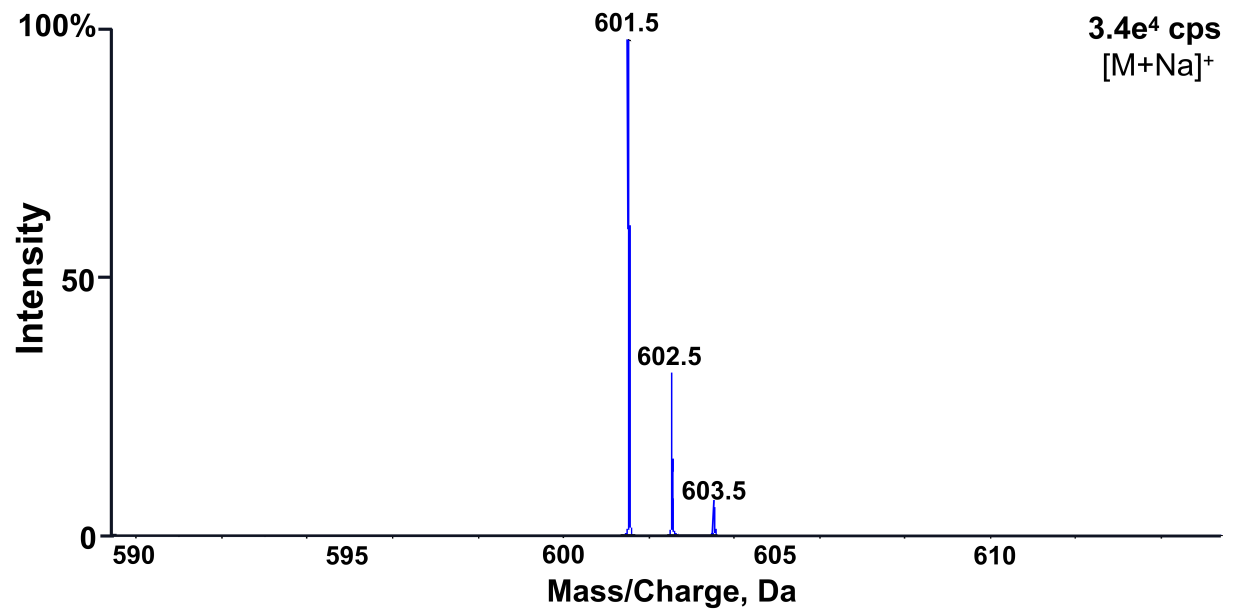


**Supplementary Figure 1. ESI-MS of 1ug/mL of DAG 16:0/18:1/0:0 as a sodium add**.


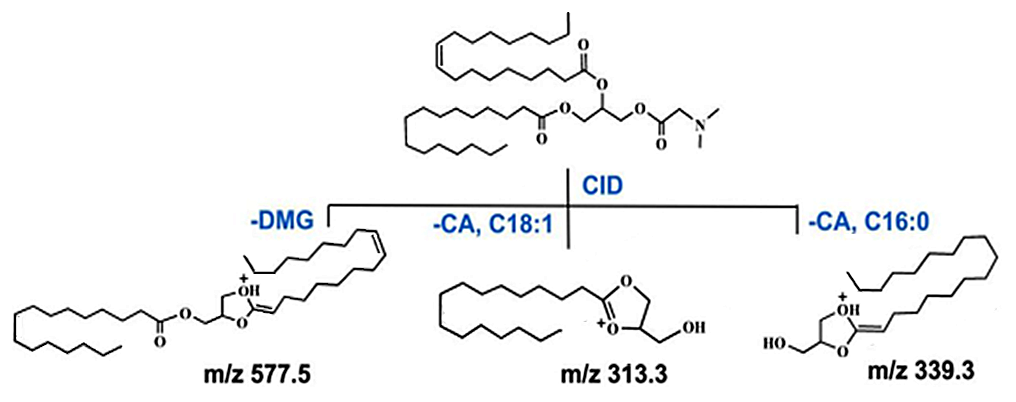


**Supplementary Figure 2.** Proposed Structures for Fragments at m/z 577, 339, and 313 resulting from CID of [ ^DMG^‑DAG 16:0/18:1/0:0]^+^


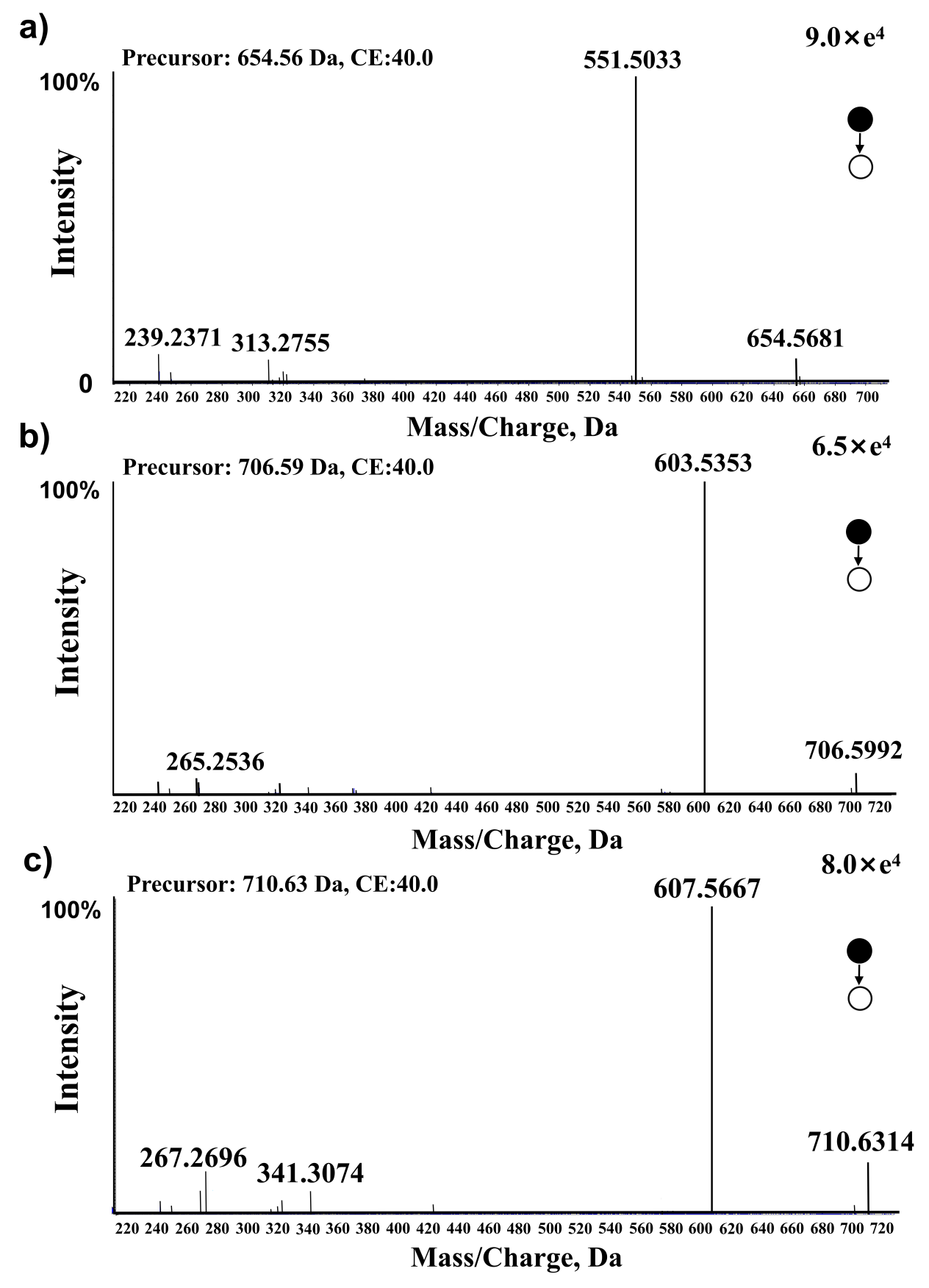


**Supplementary Figure 3.** The major fragments resulting from CID of ^DMG^-DAGs in serum samples. (a) DAG16:0/16:0/0, (b) DAG18:1/18:1/0, (c) DAG18:0/18:0/0.


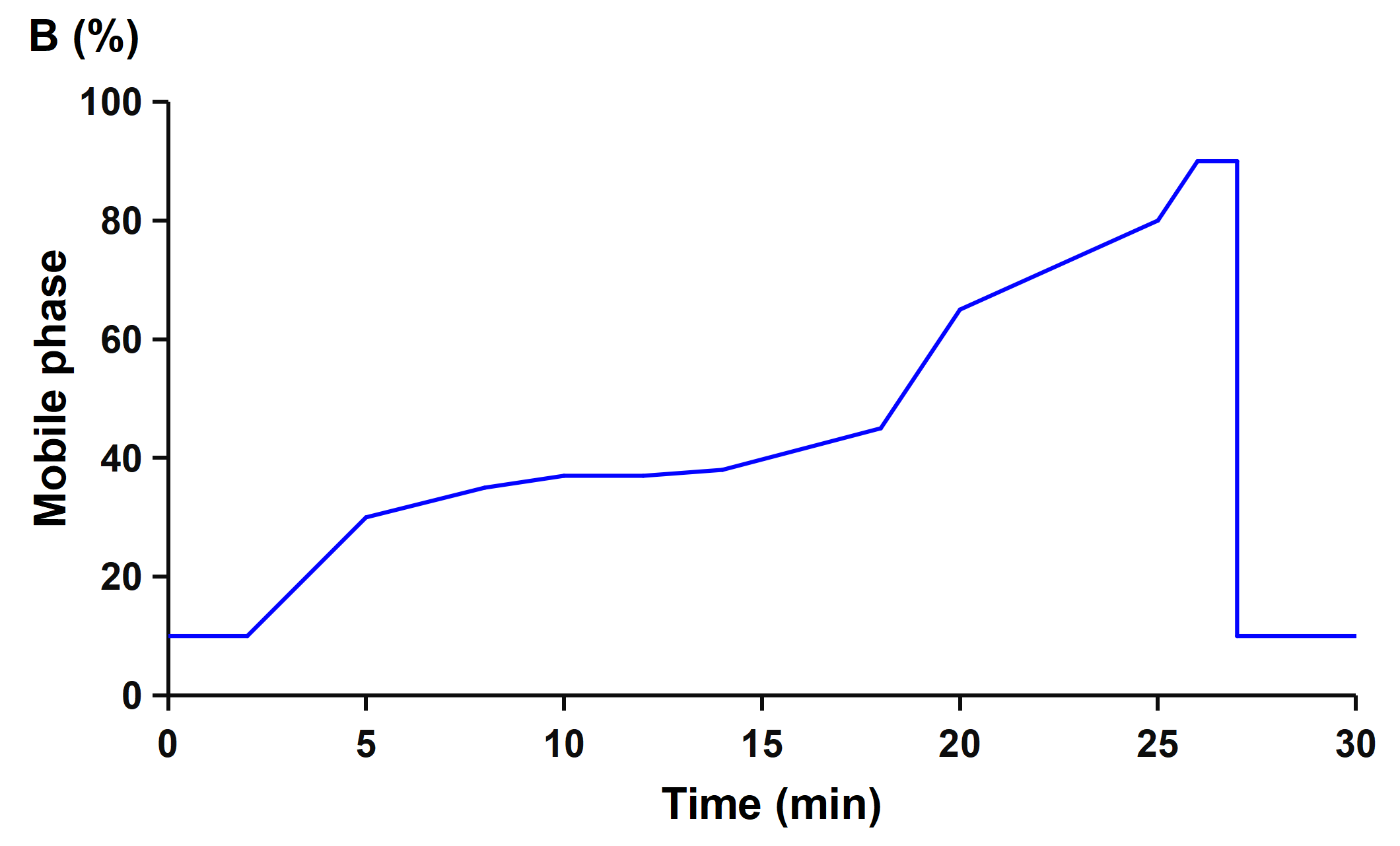


**Supplementary Figure 4.** Mobile phase gradient elution procedure for UPLC analysis

The mobile phase was delivered at a flow rate of 0.2 mL/min. The gradient program used was as follows: 10% B in 0-2 min, 10% B to 30% in 2-5 min, 30% B to 35% in 5-8 min, 35% B to 37% in 8-10 min, 37% B to 37% in 10-12 min, 38% B to 38% in 12-14 min, 38% B to 45% in 14-18 min, 45% B to 65% in 18-20 min, 65% B to 80% in 20-25 min, 80% B to 95% in 25-26 min, 95% B in 26-27 min, 10% B in 27-30 min. Source temperature of 550 °C and Spray voltage of 5.5 kV were employed for the TOF-MS analyses. For DAGs analysis, the declustering potential was set to 50 V, the collision energy was set to 40 V, the curtain gas was set to 35 psi, the drying gas was set to 55 psi, and nebulizer gas was set to 55 psi.


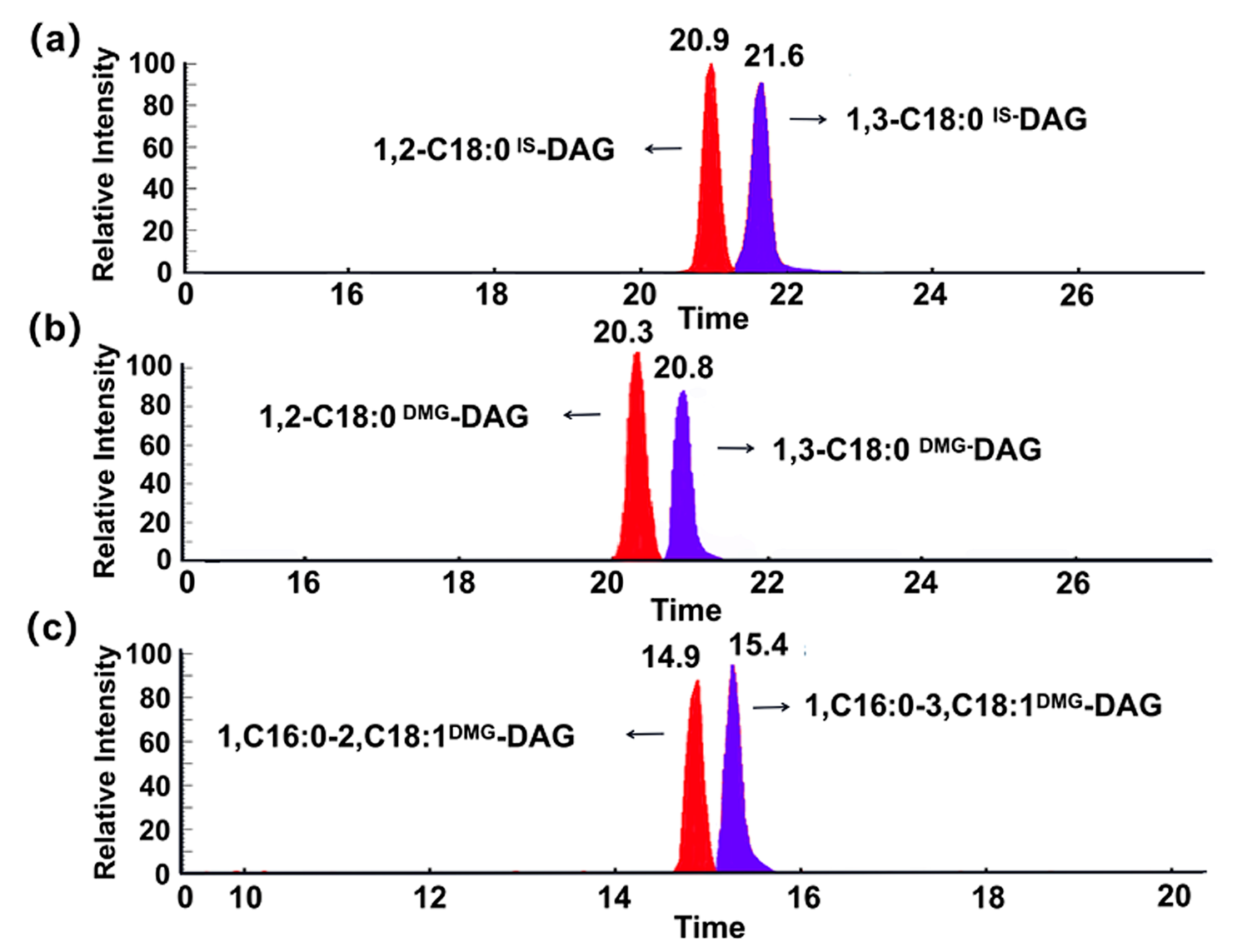


**Supplementary Figure 5.** The chromatogram of isomers DAGs, showing separation of *sn*-1,2 DAGs and *sn*-1,3 DAGs. UPLC-MS/MS chromatogram of (a) DAG 18:1/18:1/0 and DAG 18:1/0/18:1; (b) DAG 16:0/18:1/0 and DAG 16:0/0/18:1; (c) DAG 18:0/18:0/0 and DAG 18:0/0/18:0.


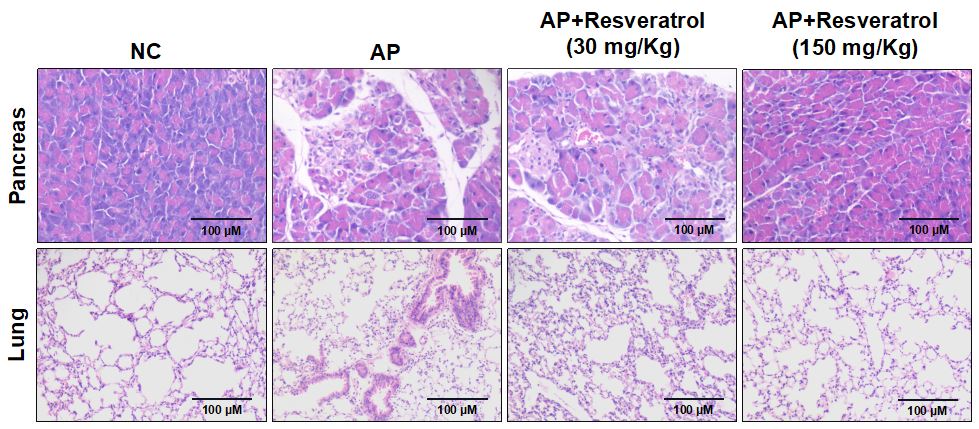


**Supplementary Figure 6.** The representative photomicrographs of H&E stained pancreas and lung tissue sections of control mice, mice pretreated with the L-arginine (3.0 g/kg and1 h apart) induction of AP, and mice pretreated with resveratrol (low-dose and high-dose) and L-arginine.

**Supplementary Table 1. Mass spectrometry quantitative parameters**

| DAGs | MRM (m/z) | Retention time (min) | EV (V) | CE (eV) | Type |
| --- | --- | --- | --- | --- | --- |
| 1,2-C8:0 | 430.0 > 327.0 | 6.21 | 21 | -22 | Quantifier |
| IS1,2-C8:0 | 444.0 > 327.0 | 6.30 | 21 | -23 | Internal standard |
| 1,2-C10:0 | 486.0 > 383.0 | 7.19 | 20 | -23 | Quantifier |
| IS1,2-C10:0 | 500.0 > 383.0 | 7.25 | 21 | -23 | Internal standard |
| 1,2-C12:0 | 542.0 > 439.0 | 8.42 | 31 | -21 | Quantifier |
| IS1,2-C12:0 | 556.0 > 439.0 | 8.54 | 31 | -22 | Internal standard |
| 1,2-C14:0 | 598.2 > 495.0 | 10.51 | 22 | -27 | Quantifier |
| IS1,2-C14:0 | 612.0 > 495.0 | 10.71 | 23 | -25 | Internal standard |
| 1,2-C16:0 | 654.0 > 551.0 | 13.77 | 29 | -29 | Quantifier |
| IS1,2-C16:0 | 668.0 > 551.0 | 13.90 | 30 | -25 | Internal standard |
| 1,2-C18:0 | 710.0 > 607.0 | 20.30 | 48 | -30 | Quantifier |
| IS1,2-C18:0 | 724.0 > 607.0 | 20.96 | 49 | -30 | Internal standard |
| 1,3-C18:0 | 710.0 > 607.0 | 20.90 | 48 | -30 | Quantifier |
| IS1,3-C18:0 | 724.0 > 607.0 | 21.65 | 48 | -30 | Internal standard |
| 1,2-C18:1 | 706.0 > 603.0 | 14.10 | 22 | -23 | Quantifier |
| IS1,2-C18:1 | 720.0 > 603.0 | 14.51 | 25 | -35 | Internal standard |
| 1,3-C18:1 | 706.0 > 603.0 | 14.60 | 22 | -23 | Quantifier |
| IS1,3-C18:1 | 720.0 > 603.0 | 14.95 | 25 | -35 | Internal standard |
| 1, C16:0-2, C18:1 | 680.0 > 577.0 | 14.34 | 35 | -29 | Quantifier |
| IS1, C16:0-2, C18:1 | 694.0 > 577.0 | 14.85 | 35 | -25 | Internal standard |
| 1, C16:0-3, C18:1 | 680.0 > 577.1 | 14.90 | 35 | -29 | Quantifier |
| IS1, C16:0-3, C18:1 | 694.0 > 577.1 | 15.40 | 35 | -25 | Internal standard |

**Supplementary Table 2.** Matrix effect and Recovery for the analysis of DAG16:0/16:0/0, DAG18:0/18:0/0, DAG18:0/0/18:0 and DAG16:0/18:1/0

| Analyte | IS-relative ME (%) | | | | | | Recovery (%, n=3) | | | | | |
| --- | --- | --- | --- | --- | --- | --- | --- | --- | --- | --- | --- | --- |
|  | Low^a^ | | Middle | | High | | Low | | Middle | | High | |
|  |  |  |  |  |  |  |  |  |  |  |  |  |
|  | 94.3 | 4.0 | 86.3 | 4.8 | 108.2 | 5.0 | 94.7 | 4.9 | 82.0 | 1.4 | 106.0 | 1.8 |
|  | 87.8 | 2.5 | 90.5 | 1.4 | 95.5 | 2.9 | 86.1 | 3.4 | 87.5 | 4.1 | 90.7 | 2.5 |
|  | 82.9 | 2.6 | 85.1 | 3.2 | 97.2 | 3.9 | 87.9 | 3.3 | 97.2 | 7.1 | 90.7 | 3.5 |
|  | 94.9 | 2.1 | 89.3 | 3.0 | 90.3 | 7.1 | 90.8 | 4.7 | 95.0 | 4.4 | 97.5 | 4.9 |

^a^ For 1,2-C16:0, the low, middle, and high concentrations were 5, 50 and 150 ng/mL, respectively; for 1,2-C18:0 and 1,3-C18:0, the low, middle, and high concentrations were 5, 25, and 100 ng/mL, respectively; for 1, C16:0-2, C18:1, the low, middle, and high concentrations were 5, 50, and 150 ng/mL, respectively.

| Analyte | Repeatability (%) | | | Stability (4°C) | |
| --- | --- | --- | --- | --- | --- |
|  | Low^a^ | Middle | High | QC(Middle) | IS(Middle) |
|  |  |  |  |  |  |
|  | 10.9 | 3.4 | 3.6 | 5.9 | 6.2 |
|  | 11.9 | 5.6 | 3.6 | 2.7 | 3.5 |
|  | 4.8 | 5.7 | 7.1 | 5.8 | 2.6 |
|  | 4.3 | 8.6 | 2.6 | 6.6 | 7.1 |

**Supplementary Table 3.** Repeatability and stability for the analysis of DAG16:0/16:0/0, DAG18:0/18:0/0, DAG18:0/0/18:0 and DAG16:0/18:1/0

^a^ For 1,2-C16:0, the low, middle, and high concentrations were 5, 50 and 150 ng/mL, respectively; for 1,2-C18:0 and 1,3-C18:0, the low, middle, and high concentrations were 5, 25, and 100 ng/mL, respectively; for 1, C16:0-2, C18:1, the low, middle, and high concentrations were 5, 50, and 150 ng/mL, respectively.
